# A case report of IPEX syndrome in Palestine: detailed family identification and breadth of disorders with the same defect

**DOI:** 10.3389/fped.2024.1438816

**Published:** 2024-09-20

**Authors:** Lana Malhis, Zeidan AbdalSalam, Yumna Njoum, Anan Abdelhaq, Muna Sharaf

**Affiliations:** ^1^ Faculty of Medicine, Najah National University, Nablus, Palestine; ^2^ Faculty of Medicine, Al-Quds University, Jerusalem, Palestine; ^3^ Department of Pediatric Endocrinology, Faculty of Medicine, Najah National University, Nablus, Palestine

**Keywords:** neonatal diabetes, diarrhea, IPEX syndrome, genotype-phenotype correlation, seizure, hypoglycemia

## Abstract

Immune dysregulation, polyendocrinopathy, enteropathy, X-linked (IPEX) syndrome is a monogenic disorder characterized by multi-systemic autoimmunity secondary to loss-of-function mutations in the gene coding the forkhead box P3 (FOXP3) transcription factor which is important for the development, maturation, and maintenance of CD4 + regulatory T (T-reg) cells. Fewer than 300 affected individuals have been identified worldwide. The occurrence of IPEX is below 1:1,000,000. Herein we present a case of a 15-day-old male who was admitted to NICU 15 days after delivery due to respiratory distress. He was found to have metabolic acidosis due to DKA. During his stay in the NICU, he experienced seizures and was intubated for a month. He was diagnosed with neonatal diabetes. He also experienced recurrent respiratory infections and multiple episodes of diarrhea rash, and meningitis. At the age of 7 months, genetic testing confirmed IPEX with FOXP3 mutation, specifically the p.(Pro75Leu) variant of the FOXP3 gene. Subsequently, multiple family members were diagnosed. The unique variability observed in organ involvement and presentation timing among individuals within the same family, despite carrying an identical mutation, is a distinctive aspect, particularly considering the monoallelic expression of the FOXP3 gene in males. This phenomenon strongly suggests the presence of modifying genes that play a significant role in the pathogenesis of IPEX syndrome. The case presentation underscores the importance of clinical suspicion of IPEX in cases of neonatal DM. It also highlights the challenges associated with managing rare genetic disorders in pediatric patients. It also emphasizes that the IPEX genotype has a wide phenotype. This case is considered the first documented case of IPEX in Palestine.

## Introduction

IPEX (immune dysregulation, polyendocrinopathy, enteropathy, X-linked), is a rare syndrome approximately 300 cases reported worldwide (1) is characterized by multi-immune phenomena, typically beginning in the first year of life. Presentation is most commonly the clinical triad of watery diarrhea, endocrinopathy (most commonly insulin-dependent diabetes mellitus), and eczematous dermatitis (2).

Nonetheless, cases reported in the last 20 years demonstrated that the IPEX clinical spectrum encompasses more than the classical triad of early-onset intractable diarrhea, type 1 diabetes, and eczema. Atypical cases of IPEX include patients with late-onset symptoms, single-organ involvement, mild disease phenotypes, or rare clinical features (3). Fetal presentation of IPEX includes hydrops, echogenic bowel, skin desquamation, IUGR, and fetal akinesia. Most children have other autoimmune phenomena including cytopenias, autoimmune hepatitis, or nephropathy; lymphadenopathy, splenomegaly, alopecia, arthritis, and lung disease related to immune dysregulation have all been observed (1). Despite the clinical heterogeneity, the unifying feature of IPEX is a mutation of the FOXP3 gene, which encodes a transcription factor essential for the maintenance of thymus-derived regulatory *T* cells (Tregs)[2]. FOXP3 is required for regulatory T (Treg)-cell development and its overexpression impaired normal CD4+ *T*-cell development and increased the frequency of CD4+ and CD8+ with suppressive function (4).

Genetic testing, either by single gene testing or multiple gene models using FOXP3 and other genes in the differential diagnosis of the disease, is necessary to establish the diagnosis (2).

Manifestations in this certain case were Diarrhea, DM, and Rash. The classic triad of the IPEX Syndrome is immune dysregulation, polyendocrinopathy, and enteropathy.

## Case presentation

A 15-day-old full-term male infant, a product of normal vaginal delivery with an uneventful pregnancy was completely well until 15 days after birth when he presented with respiratory distress necessitating intubation. He was subsequently admitted to the Neonatal Intensive Care Unit (NICU). The patient, of Palestinian ethnicity, exhibited hyperglycemia and severe diabetic ketoacidosis (DKA), prompting initial consideration of a metabolic disorder. At the time of the presentation, the patient's serum glucose level measured around 962 mg/dl, leading to the diagnosis of neonatal diabetes.

**Figure 1:**
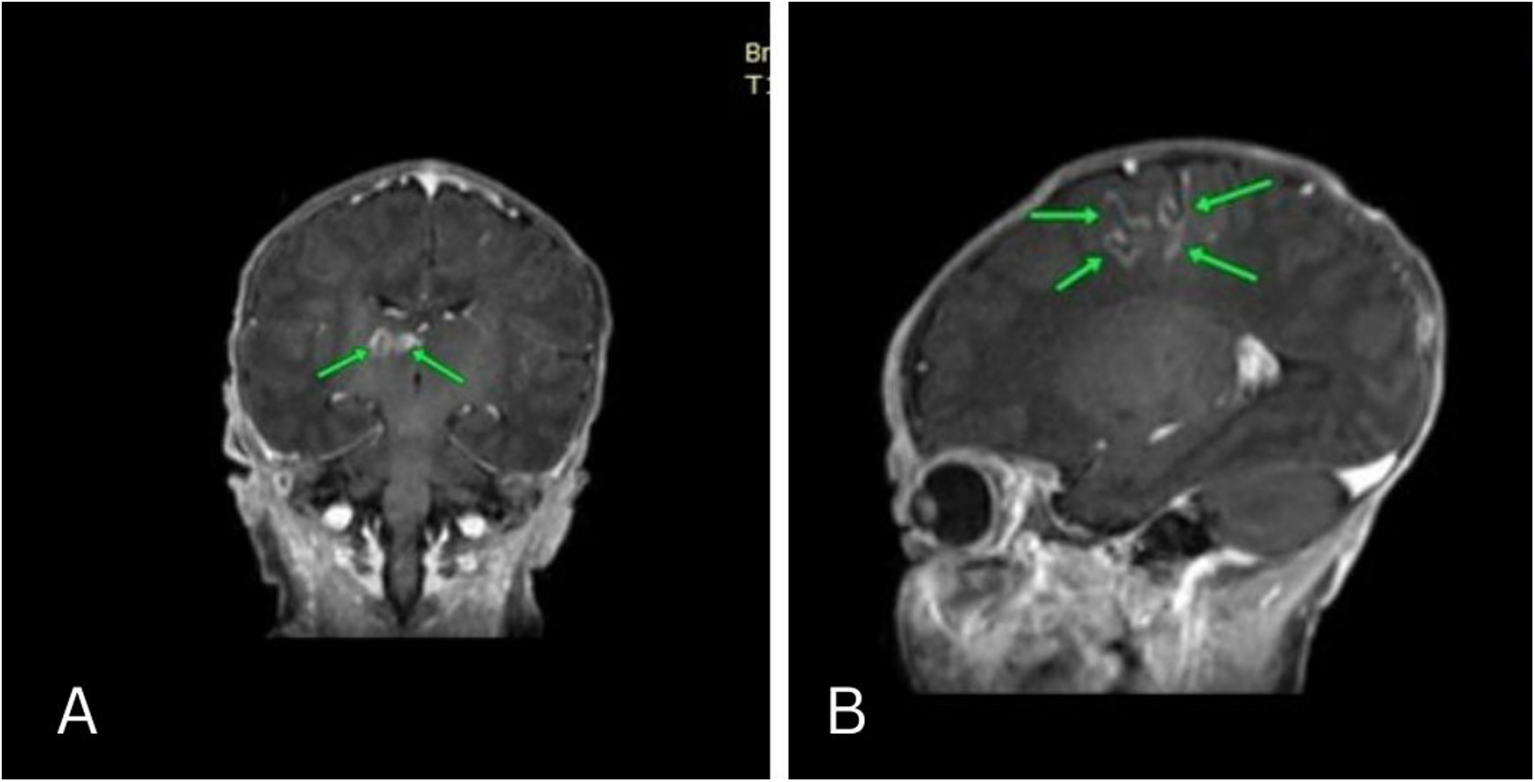
Coronal and Sagittal MRI revealed cortical areas of diffusion restriction in the left frontal and parietal lobes, displaying hyperintense T1 weighted curvilinear signal and gyral enhancement. These findings suggested subacute infarcts with cortical laminar necrosis. Additionally, two small adjacent lesions of abnormal signal intensity in the right thalamus were observed, featuring hypointensity on T1 weighted images with a hyperintense rim and faint peripheral enhancement.

Approximately 10 days into the NICU stay, the patient experienced a right-sided seizure. Lumbar puncture was done to rule out infectious origin which manifested free results. Toxoplasma and cytomegalovirus antibody tests were negative. Brain MRI revealed cortical areas of diffusion restriction in the left frontal and parietal lobes, displaying hyperintense T1 weighted curvilinear signal and gyral enhancement. These findings suggested subacute infarcts with cortical laminar necrosis. Additionally, two small adjacent lesions of abnormal signal intensity in the right thalamus were observed, featuring hypointensity on T1 weighted images with a hyperintense rim and faint peripheral enhancement. These radiological manifestations indicated potential subacute infarction with laminar necrosis, otherwise normal brain parenchyma, and cerebrospinal fluid spaces (Figure 1). These neurological and MRI findings were most likely related to ketoacidosis induced brain edema. Neurological examination at the time of delivery revealed normal findings. Subsequent physical exams during infancy were normal except for the neurological symptoms related to seizures and mild motor delay.

**Figure 2:**
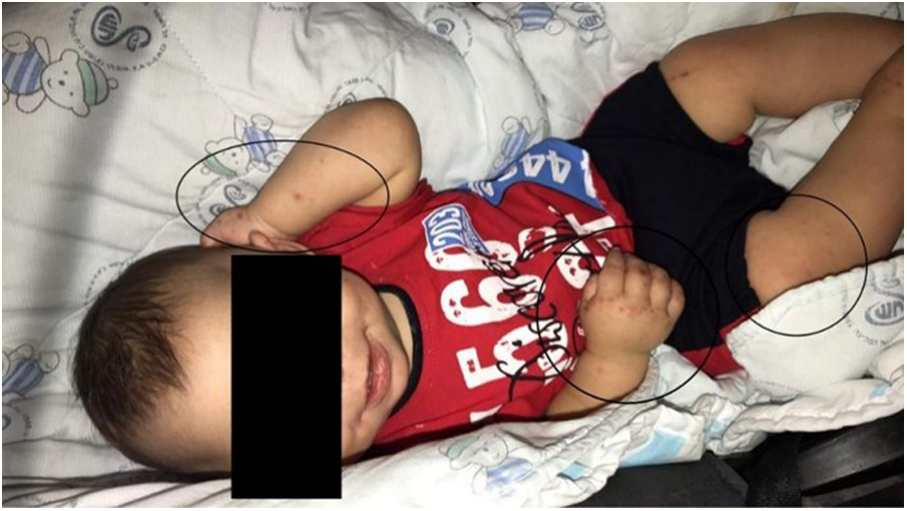
Skin rash, affecting both the trunk and limbs.

Seizure management involved a 4-month duration of phenobarbital administration. An electroencephalogram (EEG) conducted at 7 months of age yielded normal findings. Throughout infancy, the patient experienced recurrent respiratory infections necessitating multiple hospital admissions. Concurrently, episodes of watery diarrhea occurred up to five times a day without blood or foul odor. Furthermore, the patient presented with recurrent episodes of skin rash at the age of 4 months, affecting both the trunk and limbs (Figure 2). The skin rashes were characterized by erythematous, eczematous patches and plaques with occasional vesiculation and crusting. These lesions were intensely pruritic and were distributed symmetrically on the extensor surfaces of the arms and legs, as well as on the trunk. He also experienced recurrent bacterial meningitis. Considering these clinical events and a comprehensive family history, a referral was made to a tertiary specialist hospital for neurometabolic assessment.

On physical examination, the infant weighed 3.2 kg (10th percentile), length of 50 cm (25th percentile), and head circumference of 35 cm (25th percentile). He appeared lethargic and dehydrated. Respiratory examination revealed tachypnea with subcostal retractions. Auscultation of the lungs indicated bilateral crackles. Cardiovascular examination showed a regular heart rate with no murmurs. Abdominal examination was unremarkable. Neurological examination revealed hypotonia and a diminished Moro reflex.

**Figure 3:**
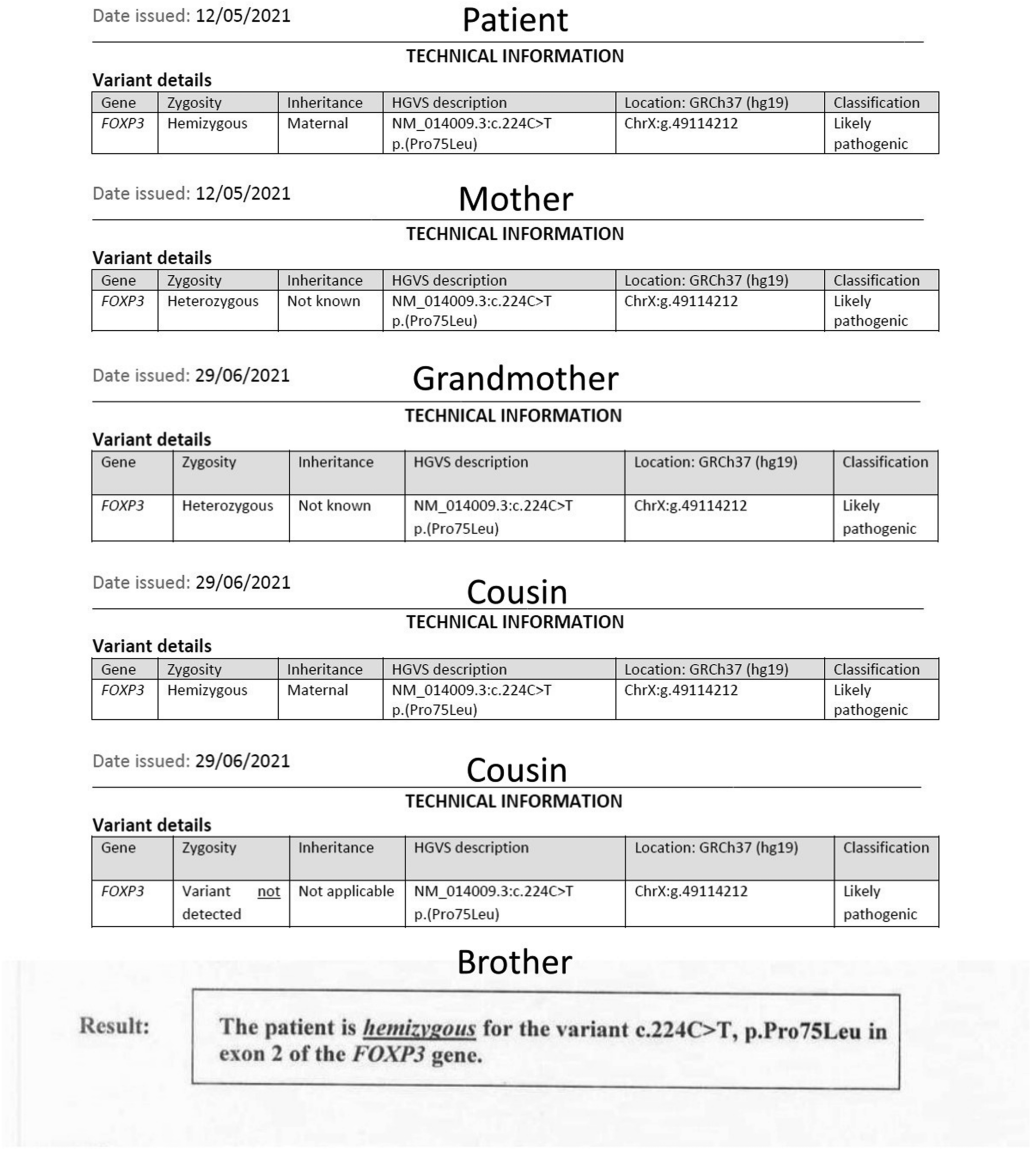
Genetic study reports of the patient and family.

Following a thorough evaluation, genetic testing was conducted at seven months of age to explore potential genetic mutations, specifically subtype FOXP3. The genetic testing revealed a mutation in p.(Pro75Leu), indicating that the patient is hemizygous for a likely pathogenic FOXP3 missense variant (Figure 3). Pathogenic variants in FOXP3 are recognized as causative factors in X-linked neonatal diabetes with associated autoimmune features, ultimately confirming the diagnosis of Immunodysregulation Polyendocrinopathy Enteropathy X-linked (IPEX) syndrome.

**Figure 4:**
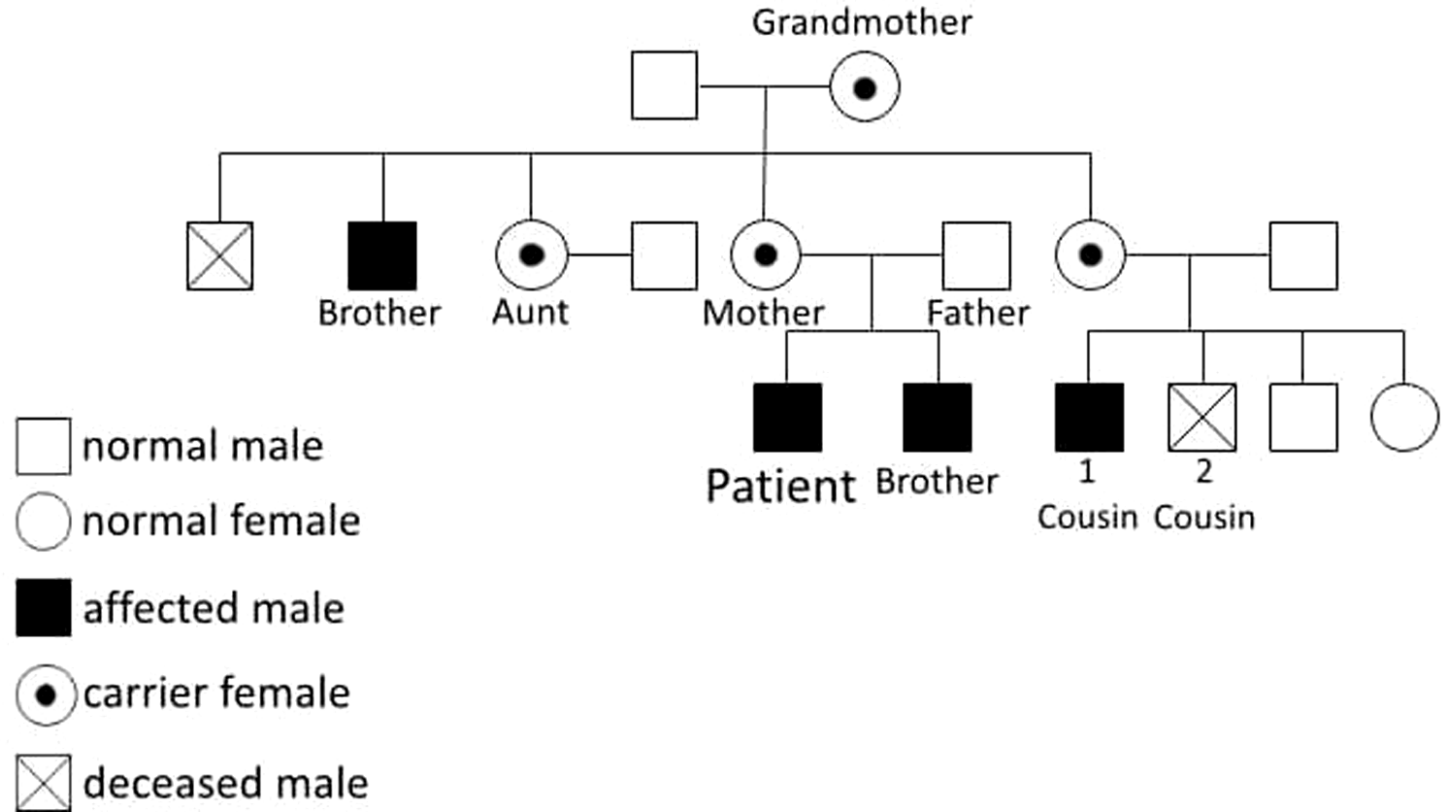
Family's Bidegree.

The family history analysis revealed significant medical complexities among close relatives. The mother remains asymptomatic, but a maternal cousin (cousin number 2 in Figure 3) has been diagnosed with insulin-dependent diabetes mellitus (IDDM) since the age of 16 months. This individual's medical history is marked by lymphadenopathy, hepatosplenomegaly, and recurrent blood transfusions due to anemia, the etiology of which is suggestive of a potential autoimmune or hemolytic origin. Another cousin (number 1 in Figure 3) within the family had a history of neonatal diabetes mellitus, diagnosed at 6 months of age, and unfortunately succumbed at 6 years old following severe diabetic ketoacidosis (DKA) complicated by cerebral edema. Furthermore, the patient's 20-year-old uncle has a medical history encompassing both rheumatoid arthritis and uncontrolled IDDM due to noncompliance since the age of 6 months. Additionally, a grandmother in her 50s has recently received a diagnosis of diabetes mellitus (DM) managed with insulin, and rheumatoid arthritis, and her screening genetic testing was positive for IPEX syndrome as a carrier. Manifestations at this age could be attributed to X-inactivation while aging. The younger brother of the patient, who is now 2 years old, tested positive for the screening test of mutation but he has no symptoms. Remarkably, genetic screening conducted among affected family members disclosed a common genetic mutation, specifically the p.(Pro75Leu) variant of the FOXP3 gene. This shared genetic anomaly may elucidate the underlying etiology and commonality of the observed medical conditions within the family. Moreover, the patient's aunt has a history of gestational diabetes, with recurrent abortion contributing further to the familial spectrum of metabolic disorders. The intricate interplay of genetic factors, as evidenced by the identified FOXP3 mutation, underscores the significance of genetic screening in comprehending the hereditary basis of these diverse medical conditions within the family (Figures 3, 4).

The patient underwent regular baseline blood tests which were normal. His last thyroid function tests were normal. Celiac antibodies including anti-tissue-transglutaminase igG and igA, anti-gliadin igA, and anti-deamidated gliadin protein epitopes igA were negative. Immunoglobulin levels were normal except for a mild decrease in IgA levels.

Many tests were sent to be performed but due to the political circumstances were unfortunately not done including complement levels, FOXP3 protein expression, FOXP3 Tregs levels, T-cell subsets, and anti-smooth muscle antibodies (Table 1).

**Table 1:** Patient's laboratory blood test results and reference ranges.

Blood index	Laboratory result	Reference range
White blood cells	8.4	(5.5–15.5) k/ul
HGB	10.7	(10.5–14) g/dl
Platelet count	360	(150–450) k/ul
Eosinophils	–	(1–3)
Basophils	–	(0–.75)
Lymphocytes	4.8	(.7–4.8) k/Ul
IgA	34	(18–171) mg/dl
IgM	34	(63–298) mg/dl
IgG	822	(507–1,407) mg/dl
IgE	12.7	(<90) mg/dl
TSH	3.9	(.6–4.9) µIU/ml
Free t4	1.04	(1.12–1.67) ng/dl
HbA1c	9	0–6.4

The patient was treated under the care of a pediatric endocrinologist and a dietitian. He was placed on insulin therapy, using Levemir or Lantus as long-acting basal insulin and Novorapid as short-acting insulin. Additionally, a diabetic-friendly diet plan was implemented. The patient did not undergo Hematopoietic Stem Cell Transplantation (HSCT) likely because diabetes was already present after the disease diagnosis, and disease manifestations were not severe enough to justify the benefit-risk balance for HSCT.

During his NICU stay, the patient received antibiotics to treat recurrent respiratory infections. The specific organisms identified during these infections included Streptococcus pneumoniae and Haemophilus influenzae. The patient was also treated for bacterial meningitis with appropriate antibiotics based on culture sensitivities.

Upon stabilization and management of his symptoms, the patient was discharged from the hospital at the age of 8 months. He is now developing well with mild motor developmental delay (walking started at age 16 months). He was provided with a comprehensive care plan that included regular follow-up visits with a pediatric endocrinologist, dietitian, and other relevant specialists to monitor his condition and manage any potential complications. The parents were educated on the administration of insulin, monitoring of blood glucose levels, and dietary management to ensure optimal care at home.

## Discussion

Different domains of FOXP3 can be affected, these mutations can be determined by sequence analysis, and their effect on protein expression is estimated using the nomenclature recommended by HGVS. Domains include the forkhead domain, repressor domain, leucine-zipper domain, zine finger domain, and the first canonical poly A sequence downstream of the coding region. Each domain has a specific cluster of disorders (5).

Diagnosis is suggested in a male patient with the clinical triad and a positive family history which was the case in the case presented in the study.

About 50% of patients with IPEX syndrome present with diabetes with onset as early as the first month of life (4). Our patient presented DKA as the first presentation of neonatal diabetes on his 15th day of life. Around 77% of patients present with T1D, followed by enteropathy approximately 6 months later. Skin manifestation completes the well-known triad of IPEX syndrome symptoms. Seizure is an uncommon manifestation with IPEX, but our patient had a seizure early in his course and was managed by phenobarbital for 4 months duration (4).

Affected females have not been reported. Heterozygous females are generally healthy, but few exceptions were observed in the form of monogenic diabetes and recurrent miscarriage. Also a negative family history doesn't preclude the diagnosis.

For the diagnosis to be established genetic testing is required through single gene testing or multiple gene models that include FOXP3 and other genes in the differential diagnosis of the disease (2). Genetic testing was done in our patient and revealed a mutation in p.(pro 75Leu) variant of the FOXP3 gene. Cases mentioned in the literature described patients with mutations in different domains of FOXP3; A child from Serbia with IVS7 + 5G>A mutations in the FOXP3 gene presented by the end of his first month by type 1 diabetes and chronic diarrhea followed by dehydration and disordered development, later he had facial eczema and laboratory thyroiditis (6). Another child from Turkey who presented with NDM at the age of 21 days was found to have a mutation at exon 10 of FOXP3 gene c.1040G>A, p.R347H. He had no other symptoms other than neonatal diabetes (7).

If a patient has immune deficiency and hyper-IgE syndrome without the classical triad of watery diarrhea, dermatitis, and T1DM/autoimmune thyroiditis in the first months of life, pediatricians should consider several primary immune deficiency disorders with hyper-IgE syndrome: hyper-IgE syndrome (HIES) or Job syndrome, Wiskott-Aldrich syndrome, IPEX, Omenn syndrome, and atypical complete DiGeorge syndrome (8).

CD4 + CD25 + FOXP3 + regulatory *T* cells (Tregs) are essential for maintaining immune tolerance and preventing autoimmunity. These specialized T cells suppress the activation and effector functions of other immune cells through mechanisms such as cell-cell contact mediated by CTLA-4 and PD-1, secretion of anti-inflammatory cytokines like IL-10 and TGF-β, and modulation of metabolic pathways (9). Dysregulation or deficiency in Treg function, often due to mutations in the FOXP3 gene as seen in disorders like IPEX syndrome, leads to autoimmune diseases characterized by unchecked immune activation against self-antigens. Therapeutically, enhancing Treg function or inducing antigen-specific tolerance holds promise for treating autoimmune diseases and improving outcomes in transplantation.

Other tests to be performed include baseline blood tests, x-rays, thyroid function tests, urine analysis, blood glucose tests, immunoglobulin assay, complement levels, celiac and other autoimmune diseases screening, T Cell subsets, Treg protein levels.

Initial treatment is usually supportive with supplemental feeding, insulin, thyroid replacement therapy if needed, immunosuppressants, topical therapies (ointments, steroids, and immunosuppressant moisturizers).

Children with classic IPEX syndrome who get no treatment typically have poor outcomes. Within the first two years of life, poor organ function, severe malabsorption, or infection are common causes of death for infants. Long-term immunosuppressive medications do not seem to prevent mortality. However, they have extended longevity. This is due to improvements in immunosuppressive regimens.

Early bone marrow transplantation can cure IPEX. An IPEX patient's chances of survival and long-term results increase the earlier they receive a transplant, particularly if it is done before permanent organ damage takes place. For example, if thyroid disease or diabetes developed before a bone marrow transplant, it will persist post-transplant.

Although, early intervention with HSCT before or immediately after the onset of DM can rescue β-cells and remit T1D completely (10). complications with bone marrow transplants can occur including graft vs. host disease, rejection, and immunosuppressant complications. As a result, considering benefit-risk factors, our patient was not eligible for HSCT.

Some studies suggest an association between gut microbiota and Treg dysfunction autoimmune disorders including both polygenic autoimmune diseases such as MS, and monogenic autoimmune diseases such as IPEX (11). This relationship proposes that these disorders can be improved by modifications of gut microbiota through probiotics and FMT.

Gene therapy is promising. However, compared with other monogenic blood diseases, restoration of FOXP3 in IPEX syndrome presents several challenges (2). The prenatal ultrasound findings of echogenic bowel and skin desquamation are associated with a diagnosis of IPEX syndrome. Fetal tissue is then required for diagnosis (12). In our patient, prenatal ultrasound was not done but the pregnancy was uneventful. Genetic counseling is recommended to prevent IPEX syndrome from developing (2). Gene counseling was recommended for the patient's family.

The unique variability observed in organ involvement and the timing of clinical presentation among individuals within the same family, despite carrying an identical mutation, is a distinctive aspect, particularly considering the monoallelic expression of the FOXP3 gene in males. This phenomenon strongly suggests the presence of modifying genes that play a significant role in the pathogenesis of IPEX syndrome.

The indication arises that performing FOXP3 gene sequencing in males with a history of neonatal diabetes, even in the absence of autoimmune-associated conditions at present, is warranted. This proactive approach is essential for early detection and intervention, given the potential for later development of autoimmune complications associated with FOXP3 mutations.

Moreover, additional comprehensive studies are imperative to elucidate the genetic and other modifiers of the FOXP3 gene that contribute to the variable clinical severity observed within the same family. Such investigations will further enhance our understanding of the intricate mechanisms underlying the diverse manifestations of IPEX syndrome, paving the way for more targeted therapeutic strategies and personalized medical interventions. The ongoing exploration of these factors holds promise for refining our knowledge of the condition and optimizing patient care.

## Conclusion

This case report is the first documentation of Immunodysregulation Polyendocrinopathy Enteropathy X-linked (IPEX) syndrome in Palestine. The patient had the classic triad of endocrinopathy, specifically neonatal diabetes mellitus (DM), dermatitis, and diarrhea. Notably, multiple family members exhibited a wide spectrum of symptoms despite sharing the same gene mutation. The symptoms and timing of presentation varied widely, with manifestations ranging from the onset of DM at the age of 50 in the grandmother to the tragic death at the age of 6 years in an undiagnosed cousin, succumbing to diabetic ketoacidosis (DKA). This case underscores the critical role of clinical suspicion and timely genetic testing, leading to the early identification of IPEX syndrome. It also highlights the challenges associated with managing rare genetic disorders in pediatric patients. This case also emphasizes the wide genotype-phenotype correlation associated with IPEX syndrome, Further research and collaborative efforts to enhance our understanding of such conditions and optimize therapeutic interventions for improved patient outcomes. This study also highlights the importance of high index for IPEX syndrome suspension in cases of diabetes presenting early in life.

## Data Availability

The original contributions presented in the study are included in the article/Supplementary Material, further inquiries can be directed to the corresponding author.
